# Supplementary material for: Long-term evolution of preferences for conservation projects in the Seto Inland Sea, Japan: a comprehensive analytic framework
Source: PeerJ. 2018 Jul 24;6:e5366. doi: 10.7717/peerj.5366 (PMC6063218; doi:10.7717/peerj.5366)
Supplement: Supplemental Information 1 [file peerj-06-5366-s001.pdf]

Supplementary Information

Index

SI1. Hypothetical Projects

SI2. Descriptive statistics

SI3. Models for confidence intervals

SI4. Summary tables for confidence intervals

SI1. Hypothetical Projects

The respondents are presented the following explanation about the three hypothetical projects.

A variety of initiatives are currently underway throughout the Seto Inland Sea to restore natural environments that were previously lost and to protect the beautiful scenery and fine natural settings that remain. The following summaries of three environmental conservation project proposals were put together by the local news desk of the Osaka branch of the Asahi Shimbun newspaper with the cooperation of the Environment Agency, researchers from all over the Seto Inland Sea, and civic groups.

Project 1: Restoring the natural beauty of coastlines

In the city of Himeji in Hyogo Prefecture, a 250-meter stretch of coastline could be restored to close to its earlier natural state by removing a concrete seawall built for land reclamation. By removing the seawall and creating a gentle slope to the shoreline, sand would be gradually deposited from the nearby Ibo River, thereby restoring a beach to its close-to-natural state in about 10 years. Stones would also be used to create a rocky shoreline which would be planted with pine trees and coastal beach plants. In consideration of the surrounding environment, leftover earth and sand removed during the restoration work would be reused within the restoration area to create a woodland rather than transporting it elsewhere.

At the outset of this project construction would be done to return a section of the shore 250 meters wide and 150 meters deep to its natural state. However, eventually, about 4 hectares of land (an area equivalent to that of the Koshien Baseball Stadium), in principle, would have to be given or lent free of charge to the prefecture.

Including the area for this project, on the Harima Sea almost all of the more than 25,000 hectares of reclaimed shoreline has been reinforced with concrete seawalls.

Project 2: Saving seagrass/seaweed beds—the cradles of the sea

The purpose of this project would be to preserve and restore seagrass beds, creating 10 hectares of test sites in Hiroshima Bay and the Sea of Aki through an initiative to grow eelgrass by transplanting it from endangered seagrass beds.

In the Seto Inland Sea, eelgrass and a seaweed called Sargassum grow in colonies, creating beds throughout the shallow coastal waters. These seagrass and seaweed beds have helped keep the sea abundant as they provide places for fish to spawn and for juvenile fish to hide, so they are also referred to as “cradles of the sea” or “green coral reefs”.

While 22,000 hectares of seagrass and seaweed beds were found throughout the Seto Inland Sea in 1960, only 6,000 hectares remain due to continual coastal reclamation and other development. Researchers warn that unless a way to save the beds of seagrass and seaweed is found quickly, drastic changes in the Seto Inland Sea’s ecosystem may result.

The 2,000 hectares of seagrass beds found in Hiroshima Bay, one of the project sites, before World War II have been almost entirely destroyed. Moreover, 50 hectares of the largest remaining seagrass bed are currently proposed for reclamation as airport and harbor construction progresses.

Project 3: Protecting natural coastlines through the Japan National Trust

While much development has taken place in the Seto Inland Sea, some coastal areas remain in their natural state, providing habitats for important organisms. For this project, a 3-kilometer stretch of the natural coastline in the Yoshinacho area of the city of Takehara in Hyogo Prefecture, where the presence of juvenile horseshoe crabs has been confirmed, would be acquired by the Japan National Trust. The horseshoe crab has been designated a Japanese natural treasure and observation of its spawning and of its young is limited to places such as Takehara and the city of Kitsuki in Oita Prefecture. In addition to the horseshoe crab, more than 50 species of animals such as fiddler crabs and seahorses live off the Takehara coast. Meanwhile, the development of a golf course is underway on the mountain overlooking the coast, which poses an imminent risk to the local ecological system. Project 3 would be the beginning of an initiative to conserve natural coastlines so a diverse variety of organisms could continue to live there.

The National Trust concept was conceived in England. To conserve areas of natural beauty contributions were collected from the public and other sources to fund the purchase and management of land in those areas through a charitable corporation.

Please read the instructions and answer the questions that follow.

Suppose that a “Seto Inland Sea Environmental Preservation Fund” were to be established to fund these three projects to conserve and restore the sea’s natural environment. The fund would need your household to make a contribution, so we are interested in understanding whether you would agree to contribute or oppose contributing to each of these three projects. You would just be making a one-time contribution to the fund and your donation would only be used for the projects you agreed with. When answering, please take care to keep in mind that you would have that much less money available to cover your usual household living expenses. If you agree to contribute to one or more projects, you will later be asked which living expenses you would cut back on as a result of your contribution.

The contribution amount proposed for each project may be different and those amounts are not a direct reflection of anything like the project’s costs. Please make your decision whether to contribute based on your personal situation and views.

SI2. Descriptive statistics

Residence

	1998		2015	
	Frequency	Percentage	Frequency	Percentage
Coastal	397	7%	457	6%
Non-coastal	5,182	92%	6,655	92%
No response	53	1%	152	2%
Total	5,632	100%	7,264	100%

Gender

	1998		2015	
	Frequency	Percentage	Frequency	Percentage
Male	4,835	86%	5,406	74%
Female	682	12%	1,858	26%
No response	115	2%	0	0%
Total	5,632	100%	7,264	100%

Age

	1998		2015	
	Frequency	Percentage	Frequency	Percentage
10-19	87	2%	0	0%
20-29	1,784	32%	1,684	23%
30-39	2,042	36%	1,846	25%
40-49	1,107	20%	1,792	25%
50-59	396	7%	660	9%
60-69	90	2%	1,031	14%
70-	7	0%	251	3%
No response	119	2%	0	0%
Total	5,632	100%	7,264	100%

Occupation

	1998		2015	
	Frequency	Percentage	Frequency	Percentage
Company employee	3,325	59%	3,248	45%
Civil servant	565	10%	315	4%
Self-employed	254	5%	572	8%
University student	783	14%	407	6%
High school student	36	1%	2	0%
Part-time worker	73	1%	848	12%
Unemployed	77	1%	890	12%
Housewife	81	1%	843	12%

Other	294	5%	139	2%
No response	144	3%	0	0%
Total	5,632	100%	7,264	100%

Household Income

	1998		2015	
	Frequency	Percentage	Frequency	Percentage
Less than ¥2,000,000	380	7%	629	9%
¥2,000,000–¥2,999,999	175	3%	514	7%
¥3,000,000–¥3,999,999	407	7%	899	12%
¥4,000,000–¥4,999,999	449	8%	864	12%
¥5,000,000–¥5,999,999	580	10%	746	10%
¥6,000,000–¥6,999,999	572	10%	538	7%
¥7,000,000–¥7,999,999	567	10%	398	5%
¥8,000,000–¥8,999,999	519	9%	335	5%
¥9,000,000–¥9,999,999	367	7%	251	3%
¥10,000,000–¥10,999,999	571	10%	186	3%
¥11,000,000–¥11,999,999	143	3%	61	1%
¥12,000,000–¥12,999,999	179	3%	83	1%
¥13,000,000–¥13,999,999	114	2%	37	1%
¥14,000,000–¥14,999,999	80	1%	37	1%
¥15,000,000–	282	5%	115	2%
No response	247	4%	1,571	22%
Total	5,632	100%	7,264	100%

SI3. Logit models for WTP estimates with their confidence intervals

Coastal Residents

	Project 1				Project 2				Project 3			
	1998		2015		1998		2015		1998		2015	
Variables	Coefficient	t-stat.	Coefficient	t-stat.	Coefficient	t-stat.	Coefficient	t-stat.	Coefficient	t-stat.	Coefficient	t-stat.
Constant	2.2260	9.235 ***	1.9670	8.822 ***	1.8540	9.005 ***	1.8400	8.973 ***	1.7500	4.593 ***	1.9230	9.08 ***
Income									8.78E-04	2.012 **		
Bid	-1.03E-04	-6.851 ***	-1.04E-04	-7.098 ***	-1.07E-04	-7.498 ***	-9.08E-05	-6.806 ***	-1.09E-04	-8.069 ***	-1.01E-04	-7.258 ***
N	278		286		304		308		305		303	
Log-likelihood	-129.1674		-144.8370		-155.5196		-160.2309		-132.7329		-154.0420	
Pseudo-R^2	0.1766		0.1757		0.1862		0.1429		0.2392		0.1719	

Noncoastal Residents

	Project 1				Project 2				Project 3			
	1998		2015		1998		2015		1998		2015	
Variables	Coefficient	t-stat.	Coefficient	t-stat.	Coefficient	t-stat.	Coefficient	t-stat.	Coefficient	t-stat.	Coefficient	t-stat.
Constant	1.3920	13.148 ***	1.1460	11.983 ***	1.4160	14.013 ***	1.1230	12.031 ***	1.8400	17.3 ***	1.1120	11.946 ***
Income	6.93E-04	5.658 ***	6.27E-04	4.462 ***	5.05E-04	4.317 ***	5.60E-04	4.117 ***	3.81E-04	3.163 ***	6.81E-04	4.996 ***
Bid	-1.05E-04	-25.147 ***	-7.91E-05	-20.323 ***	-1.21E-04	-26.867 ***	-7.20E-05	-19.351 ***	-1.15E-04	-27.531 ***	-7.09E-05	-19.186 ***
N	3440		3146		3598		3260		3772		3320	
Log-likelihood	-1717.1390		-1772.0370		-1827.7630		-1869.6690		-1774.7420		-1875.5050	
Pseudo-R^2	0.1933		0.1211		0.2192		0.1035		0.2177		0.1026	

***$p < 0.01$; ** $p < 0.05$

SI4. Summary tables for confidence intervals

		Project 1		Project 2		Project 3	
		1998	2015	1998	2015	1998	2015
Coastal residents	Mean WTP (JPY)	19,216	17,585	16,596	18,078	19,774	17,530
	95% CI (JPY)	{17,245; 21170}	{15,692; 19,512}	{14,738; 18,580}	{16,241; 19,949}	{17,934; 21,500}	{15666; 19492}
	Income	no	no	no	no	yes	no
	N	278	286	304	308	305	303
Non-coastal residents	Mean WTP (JPY)	17,181	17,114	14,870	17,452	17,397	17,971
	95% CI (JPY)	{16,607; 17,759}	{16538; 17699}	{14,333; 15,431}	{16867; 18022}	{16,859; 17,962}	{17,409; 18,535}
	Income	yes	yes	yes	yes	yes	yes
	N	3,440	3146	3,598	3260	3,772	3320
